# Notch3 inhibits epithelial–mesenchymal transition in breast cancer via a novel mechanism, upregulation of GATA-3 expression

**DOI:** 10.1038/s41389-018-0069-z

**Published:** 2018-08-13

**Authors:** Hao-Yu Lin, Yuan-Ke Liang, Xiao-Wei Dou, Chun-Fa Chen, Xiao-Long Wei, De Zeng, Jing-Wen Bai, Yu-Xian Guo, Fang-Fang Lin, Wen-He Huang, Cai-Wen Du, Yao-Chen Li, Min Chen, Guo-Jun Zhang

**Affiliations:** 1 The Breast Center, The Cancer Hospital of Shantou University Medical College (SUMC), Shantou, China; 2 ChangJiang Scholar’s Laboratory, The Cancer Hospital of Shantou University Medical College (SUMC), Shantou, China; 3 Department of Breast and Thyroid Surgery, The First Affiliated Hospital of SUMC, Shantou, China; 4 Department of Medical Oncology, University Medical Center Groningen, University of Groningen, Hanzeplein 1, 9713 GZ Groningen, The Netherlands; 5 Department of Pathology, The Cancer Hospital of Shantou University Medical College (SUMC), Shantou, China; 6 Department of Breast Medical Oncology, The Cancer Hospital of Shantou University Medical College (SUMC), Shantou, China; 7 The Cancer Center, Xiang’an Hospital, Xiamen University Medical College, Xiang’an Dong Rd, 2000 Xiamen, China; 8 Department of Oncology, Shenzhen Hospital of Chinese Academy of Medical Science affiliated Cancer Hospital, Shenzhen, China

## Abstract

Notch3 and GATA binding protein 3 (GATA-3) have been, individually, shown to maintain luminal phenotype and inhibit epithelial–mesenchymal transition (EMT) in breast cancers. In the present study, we report that Notch3 expression positively correlates with that of GATA-3, and both are associated with estrogen receptor-α (ERα) expression in breast cancer cells. We demonstrate in vitro and in vivo that Notch3 suppressed EMT and breast cancer metastasis by activating GATA-3 transcription. Furthermore, Notch3 knockdown downregulated GATA-3 and promoted EMT; while overexpression of Notch3 intracellular domain upregulated GATA-3 and inhibited EMT, leading to a suppression of metastasis in vivo. Moreover, inhibition or overexpression of GATA-3 partially reversed EMT or mesenchymal–epithelial transition induced by Notch3 alterations. In breast cancer patients, high GATA-3 expression is associated with higher Notch3 expression and lower lymph node metastasis, especially for hormone receptor (HR) positive cancers. Herein, we demonstrate a novel mechanism whereby Notch3 inhibit EMT by transcriptionally upregulating GATA-3 expression, at least in part, leading to the suppression of cancer metastasis in breast cancers. Our findings expand our current knowledge on Notch3 and GATA-3's roles in breast cancer metastasis.

## Introduction

Breast cancer remains the most frequent cancer of female and the principal contributing to the cancer associate mortality in women all around the world^1^. Breast cancers are usually divided into those that are hormone-dependent and those that are hormone-independent according to their estrogen receptor (ER) status. The ER-positive subtype of breast cancers features cells that are sensitive to estrogen ablation, which contributes to its generally favorable prognosis. Many factors may modulate ER expression, such as GATA-3 and pS2, as well post-transcriptional modifications.

Malignant transformation of mammary epithelia is facilitated by alterations in various genes and signaling pathways, including Notch, Wnt, and GATA, that cause abnormal cellular growth and differentiation^2-5^. Among the GATA family (GATA 1–6), GATA-3 is the most abundantly expressed in luminal epithelial cells. GATA-3 is essential for the development of human mammary gland and the differentiation breast cancer cell^6–9^. For example, a loss of expression of the ERα is observed in GATA-3 knockout mice^9^. With regard to breast cancer, a positive feedback loop has been identified to affect GATA-3 and ERα regulation^6^; both GATA-3 and ERα can be used as markers to predict patient responses to endocrine treatment of breast cancer ^6^.

Elevated GATA-3 expression in breast cancer cells leads to differentiation, and thereby suppression of tumor dissemination^9^. GATA-3 is involved in the process of EMT by regulating different cellular signaling pathways^10^. Studies from Dydensborg et al. demonstrated that overexpression of GATA-3 result in inhibiting tumor growth and pulmonary metastasis by repressing inhibitor of differentiation 1 (ID1) and ID3 and induced deletion in liver cancer 1 (DCL1) and progestogen-associated endometrial protein (PAEP)^11^. Unsurprisingly, decrease or loss of expression of GATA-3 is associated with pathogenesis, hormone receptor negativity, and an unfavorable prognosis for breast cancer patients in the clinic^9,12^. Moreover, the GATA-3 gene has been identified with mutations in > 10% of all breast cancers, a distinction held by only three genes^13^. Collectively, such data indicate that GATA-3, function as a tumor suppressor, may emerge as a potential biomarker to detect and predict risk of breast cancer development.

Similar to the GATA family, Notch signaling is also fundamental to maintenance of stem cell, cellular differentiation, and determination^14^. Emerging evidence shows that Notch3 may play essential role in mammary gland development and commitment to luminal fate^15,16^. Notch3 is expressed in a luminal progenitor cell population that is highly clonogenic and transiently quiescent, and differentiates into a ductal lineage^17^. Moreover, loss of Notch3 expression reduces luminal cell production from bipotent progenitors^18^. Intriguingly, Notch 1 to 4 signaling has distinct activities and biological functions in tumorigenesis^19^. Notch2 or Notch3 signaling have been shown to suppress tumor growth through a reduction of stem cell number^20–22^, and to inhibit the tumorigenesis and metastasis of breast cancers^23^. EMT is a characteristic transformation of epithelial cells involving depolarization, loss of cell–cell contacts, and morphological change from epithelioid to fibroblastic, and is thought to confer metastatic characteristics to developing carcinomas^24^. Intriguingly, two recently published articles of our studies demonstrated that Notch3, but not other Notch receptors, is positively correlated to ERα both in breast cancer cells and breast carcinoma tissues. We also showed that Notch3 negatively regulated EMT through Kibra-mediated Hippo/YAP and ERα signaling pathway in vitro^25^. Notch3 signaling inhibits EMT either by regulating target gene expression via interactions with the nuclear CBF1/RBP-Jκ/*S*uppressor of Hairless/LAG-1 (CSL; RBP-Jκ in mice) transcriptional complex (e.g., ERα and Bmi1) or by regulating noncanonical expression of the Wnt signaling receptor frizzled7 (FZD7)^26^. These findings suggest that Notch3 signaling inhibits EMT in breast cancer cells by activating novel downstream genes.

These findings prompted us to investigate whether there is a correlation between Notch3 and GATA-3, and how they are regulated by each other, especially in ER-positive subtype breast cancer. In the present study, we present solid evidence demonstrating that activated Notch3 maintains the epithelial phenotype and suppresses metastasis through the transcriptional regulation of GATA-3 in breast cancer.

## Results

### Elevated expression of Notch3 and GATA-3 is correlated with a luminal epithelial phenotype in breast cancer cell lines

**Fig. 1:**
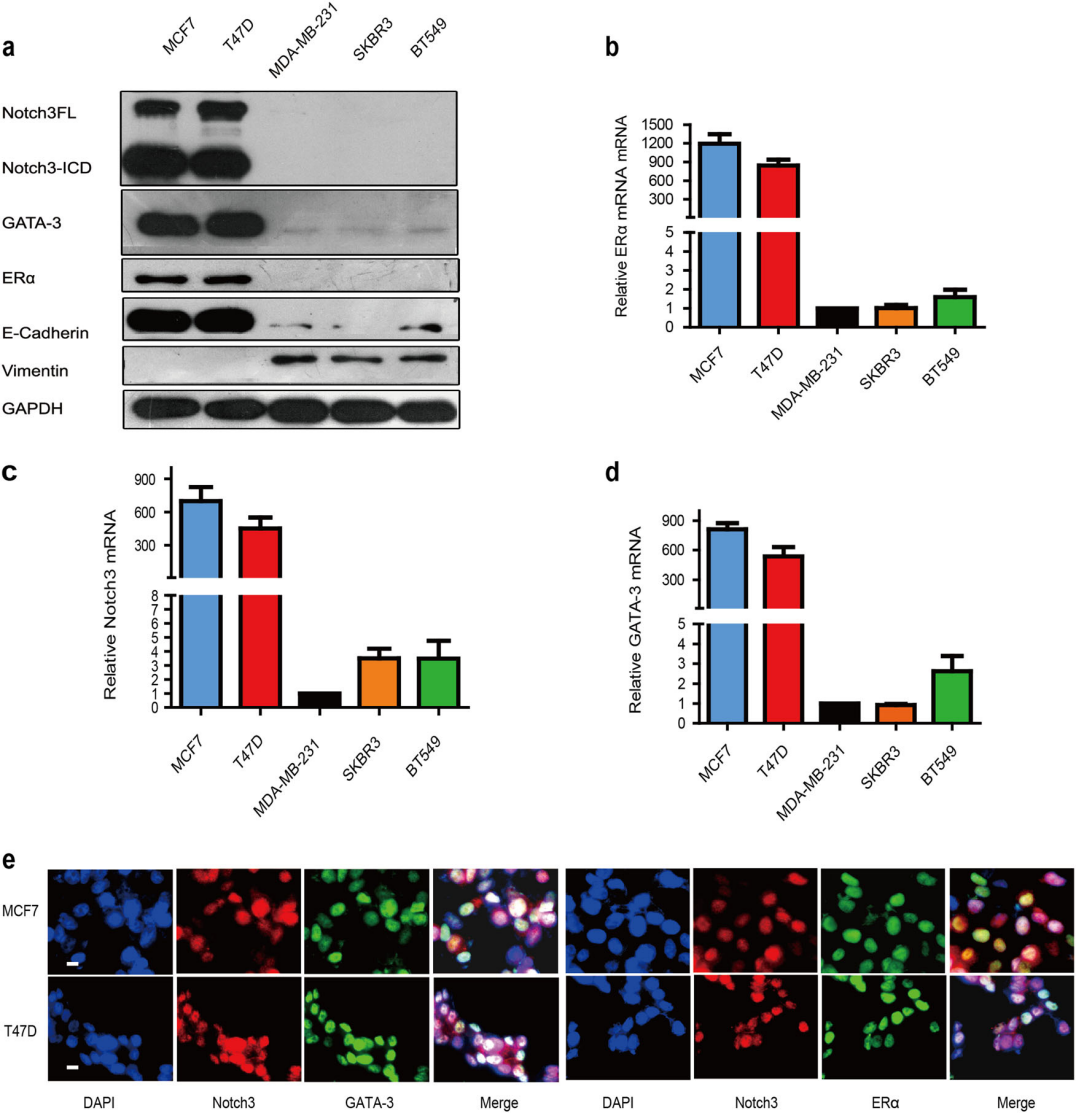
Correlation of Notch3 and GATA-3 expression in various breast cancer cell lines. **a** Expression of Notch3, GATA-3, ERα, E-cadherin, and vimentin analyzed by western blot in breast cancer cell lines. **b**–**d** Expression of Notch3, GATA-3, and ERα mRNA analyzed by reverse transcription (RT)–PCR in breast cancer cell lines. **e** Confocal fluorescence microscopy of DAPI/Notch3/GATA-3 and DAPI/Notch3/ERα staining in MCF-7 (top) and T47D (bottom) cells. The scale bar represents 200 μm

To explore the possible roles for Notch3 and GATA-3 in breast cancer, we detected their expression levels in a series of breast cancer cell lines. Both Notch3 (including full-length (FL) and intracellular domain (ICD)) and GATA-3 proteins were primarily expressed in luminal epithelial phenotype T47D and MCF-7 cells, corresponding to an increase in E-Cadherin and ERα protein expression (Fig. 1a). Significantly lower expressions of Notch3 and GATA-3 were found in ERα-negative cells, including MDA-MB-231 and BT-549 cell lines (basal-like) and in the SK-BR-3 cell line (HER2-positive), which expresses vimentin, a marker of the stromal cell phenotype (Fig. 1a). We further interrogated Notch3 and GATA-3 mRNA in several breast cancer cell lines to validate results at the protein level. Unsurprisingly, we detected high Notch3 and GATA-3 mRNA levels in MCF-7 and T47D cells (ERα-positive), but not in the MDA-MB-231, BT-549 or SK-BR-3 cells (ERα- negative) (Fig. 1b, c, d). Immunofluorescence further confirmed that Notch3 and GATA-3 are primarily expressed in MCF-7 and T47D cells. Moreover, immunofluorescence staining showed fluorescence co-localization of these two proteins in cellular nucleus of both cell lines (Fig. 1e). These results imply a possible connection between Notch3 and GATA-3 expression in luminal epithelial phenotypes of breast cancer.

### Notch3 positively regulates GATA-3 mRNA and protein expression

**Fig. 2:**
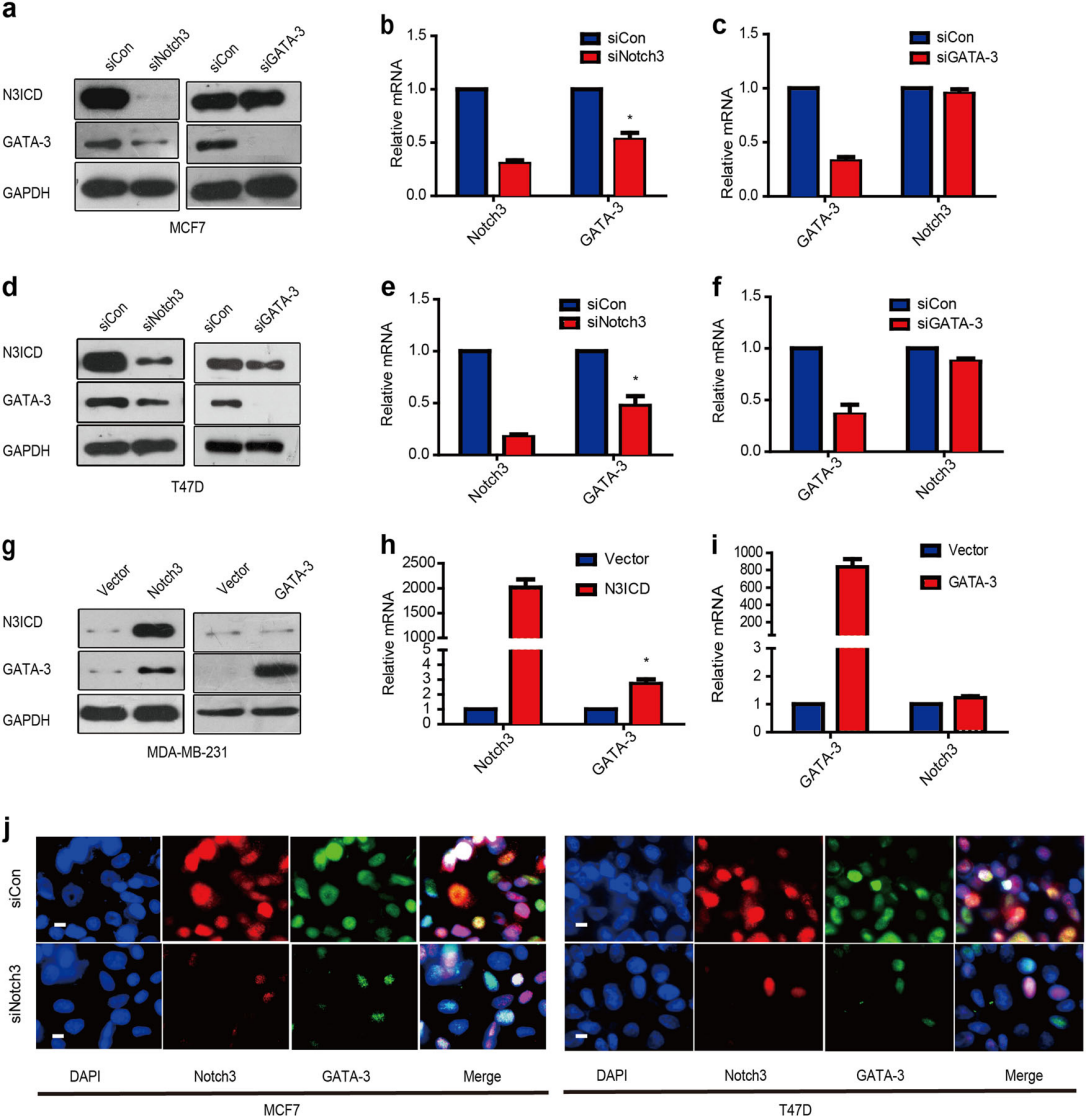
Notch3 regulates expression of GATA-3 transcriptionally. **a**–**c** Expression of Notch3 and GATA-3 in MCF-7 cells at protein and mRNA levels analyzed by western blot (**a**) and RT–PCR, respectively, **b**–**c** when silencing Notch3 or GATA-3 by siRNA (**d**–**f**). Expression of Notch3 and GATA-3 in T47D cells at protein and mRNA levels analyzed by western blot (**d**) and RT–PCR (**e**–**f**), respectively, when silencing Notch3 or GATA-3 by siRNA. **g**–**i** Expression of Notch3 and GATA-3 in MDA-MB-231 cells at protein and mRNA levels analyzed by western blot (**g**) and RT–PCR, respectively, (**h**–**i**) when overexpressing N3ICD or GATA-3. **j** Confocal fluorescence microscopy of DAPI/Notch3/GATA-3 staining in MCF-7 (left) and T47D (right) cells, treated with siControl (top) or siNotch3 (bottom). The scale bar represents 200 μm

To explore the possible regulation between these two genes, we silenced or overexpressed Notch3 or GATA-3 in breast cancer cells. Suppression of Notch3 by siRNA caused a significant downregulation of GATA-3 in ERα-positive MCF-7 and T47D cells at both the mRNA and protein levels, while suppressing GATA-3 did not affect the expression of Notch3 (Fig. 2a–f). Three different siRNA sequences were used to silence Notch3 or GATA-3 separately. It was found that siNotch3#2/siNotch3#3 and siGATA-3#1/siGATA-3#2 all showed effective downregulation of the target genes (Figure S1a–b), so we chose siNotch3#2 and iGATA-3#2 for further experiments. Conversely, enforced expression of the Notch3 ICD led to upregulation of GATA-3 mRNA and protein in MDA-MB-231 cells (Fig. 2g–i). Notch3 and GATA-3 co-localize in the nucleus of MCF-7 and T47D cells as demonstrated by immunofluorescence microscopy, but Notch3 suppression reduced GATA-3 expression (Fig. 2j). Collectively, our results suggest that GATA-3 expression is positively regulated by Notch3 in breast cancer cells. To confirm the diminished and elevated Notch signaling experiments, the expression of general Notch receptor target gene Hes1 and Hey1, also detected by real-time PCR (Figure S3a–c)

### Notch3 activates GATA-3 expression via CSL-binding elements in the GATA-3 promoter

**Fig. 3:**
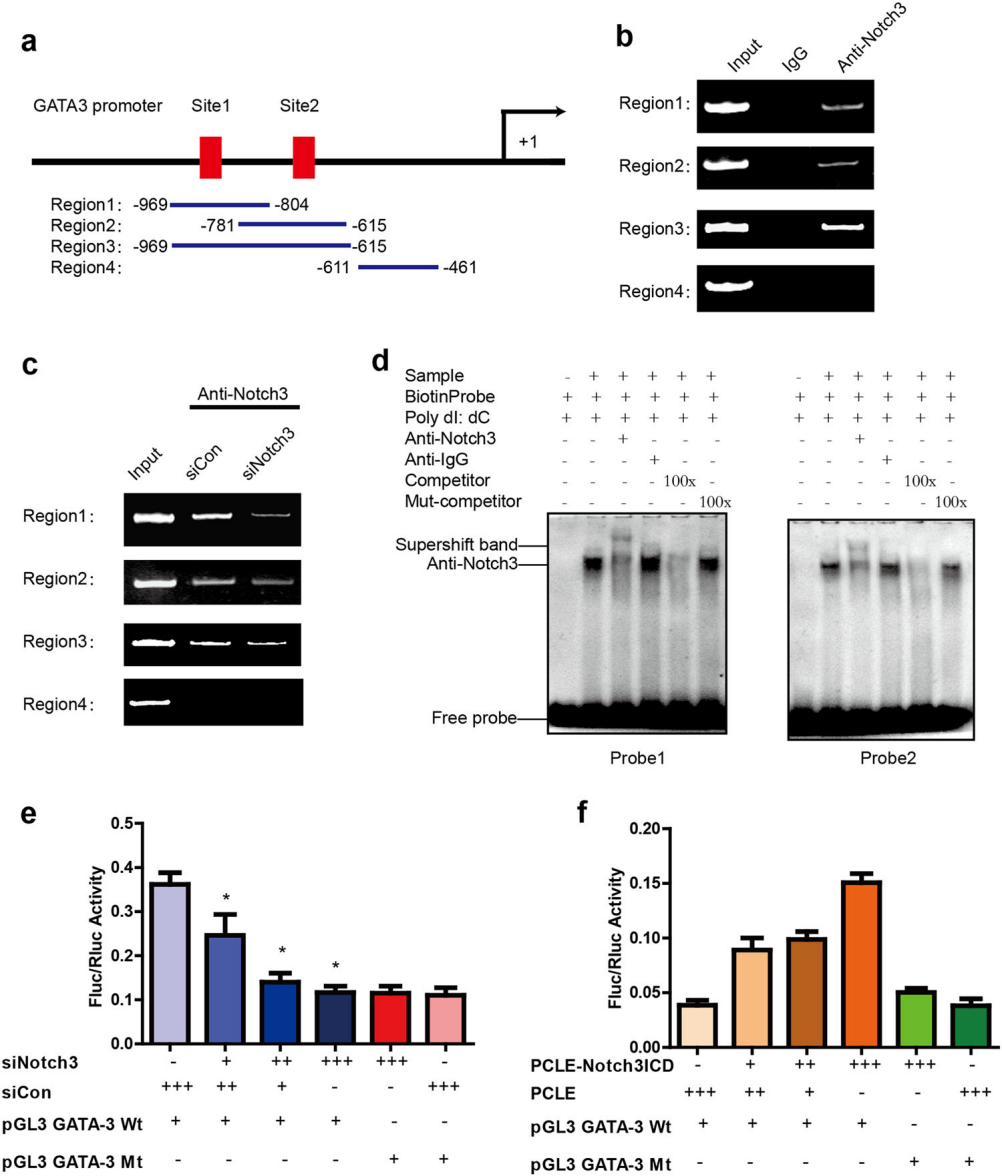
Notch3 upregulates GATA-3 expression by binding to the core element of the GATA-3 promoter. **a**–**c** Schematic representation of the three CSL-binding element-containing primers (Regions 1 and 2 containing respective single CSL-binding elements, region 3 containing both CSL-binding elements) and negative control primers (region 1 does not contain a CSL-binding element) used for ChIP assays. Bands were seen in regions 1, 2, and 3 of the PCR product but not in the negative control region 4 (**b**); bands were dramatically decreased in regions 1, 2, and 3 of the PCR product when cells were treated with siNotch3 (**c**). **d** Probes 1 and 2 represent two biotin probes containing the core element of the CSL-binding sites of the GATA-3 promoter. Supershift bands (lane 3) were seen in the presence of an anti-Notch3 antibody, but not in the presence of an anti-IgG antibody (lane 4). Competition assays were performed using a 100-fold (lane 5) excess of unlabeled oligonucleotide containing the core element of the CSL-binding element of the GATA-3 promoter. A 100-fold excess of unlabeled mutation oligonucleotide containing the mutated core element of the CSL-binding element (lane 6) was used for mutation competition assays. Nuclear extracts from MCF-7 cells were not added to lane 1. **e** Notch3 was silenced in MCF-7 cells by siRNA and then co-transfected with the GATA-3 promoter or a mutated GATA-3 promoter (deleting both CLS-binding elements) construct containing Firefly luciferase, and internal control plasmid, pRL-SV40, containing *Renilla* luciferase. Firefly luciferase/*Renilla* luciferase values were used to indicate promoter activity. Each sample was tested in triplicate. *P < 0.05. **f** N3ICD was overexpressed in MDA-MB-231 cells and co-transfected with the GATA-3 promoter or a mutated GATA-3 promoter (deleting both CSL-binding elements) construct containing Firefly luciferase and internal control plasmid, pRL-SV40, containing *Renilla* luciferase. Firefly luciferase/*Renilla* luciferase values were used to indicate promoter activity. Each sample was tested in triplicate. *P < 0.05

Notch family transcription factors regulate downstream target molecules via directly bound to CSL promoter elements. To explore whether Notch3 regulation of GATA-3 is regulated by direct binding to CSL elements within its promoter, we next carried out chromatin immunoprecipitation assays (ChIP) in MCF-7 cells using an antibody against Notch3. Two putative CSL-binding motifs were identified upstream (−829–−834 bp and −665–−670 bp) of exon 1 in the GATA-3 promoter. Three pairs of primers were designed and synthesized according to span CSL-binding elements with sequence of GGGAA^27,28^ located in upstream (Region 1: −969–−804 bp, containing CSL site 1; Region 2: −781–−615 bp, containing CSL site 2; and Region 3: −969–−615 bp, containing CSL sites 1 & 2) of exon 1 in the GATA-3 promoter. For region 4: −611–−461 bp, one pair of primers was designed to not contain any CSL-binding elements as a negative control (Fig. 3a). We observed Notch3 binding in the three regions that containing CSL-binding elements, but absent in the control region (Region 4; Fig. 3b). Furthermore, the downregulation of Notch3 by siRNA caused decreased binding of the GATA-3 promoter in the three regions, but not in Region 4 (Fig. 3c).

We next employed electrophoretic mobility shift assays (EMSAs) to determine elements required for Notch3 binding within the GATA-3 promoter (Fig. 3d). Notch3 was found to bind both core elements in the GATA-3 promoter. Competition assays added with excess unlabeled nucleotides eliminated the Notch3 shifting band; this was not observed in the presence of an excess of unlabeled, mutated oligonucleotide (TGTCT; Fig. 3d). A super-shifted band was observed after addition of labeled probe and anti-Notch3 antibody to MCF-7 nuclear extracts, demonstrating that Notch3 is capable of binding to the typical core bound element (GGGAA) of the GATA-3 promoter. Our findings show that Notch3 is a component of a complex that binds the GATA-3 promoter.

Next, the transcriptional activity involved in GATA-3 regulation by Notch3 was further evaluated. We cloned the GATA-3 promoter (approximately 1 kb) to drive luciferase expression in the reporter plasmid pGL3, and then determined luciferase activity in the presence or absence of the Notch3 intracellular (transcription activating) domain (N3ICD). In MCF-7 cells, the luciferase activity of this reporter decreased in a dose-dependent manner after suppressing Notch3 expression (Fig. 3e), while in MDA-MB-231 cells, the luciferase activity increased following enforced expression of N3ICD (Fig. 3f). Comparing the activity of wild-type and mutant GATA-3 promoters, the latter of which carried deletions of both Notch3 binding elements (GGGAA), demonstrated that luciferase activity was significantly reduced (from 30 to 70%) in wild-type reporters, in a dose-dependent manner. GATA-3 promoter activity increased 2–4 fold (Fig. 3e, f) when N3ICD was co-expressed in MDA-MB-231 cells. These results imply that Notch3 drives GATA-3 promoter activity by directly binding to CSL-binding elements.

### Notch3 knockdown promotes EMT in MCF-7 cells, which is attenuated by overexpressing GATA-3

**Fig. 4:**
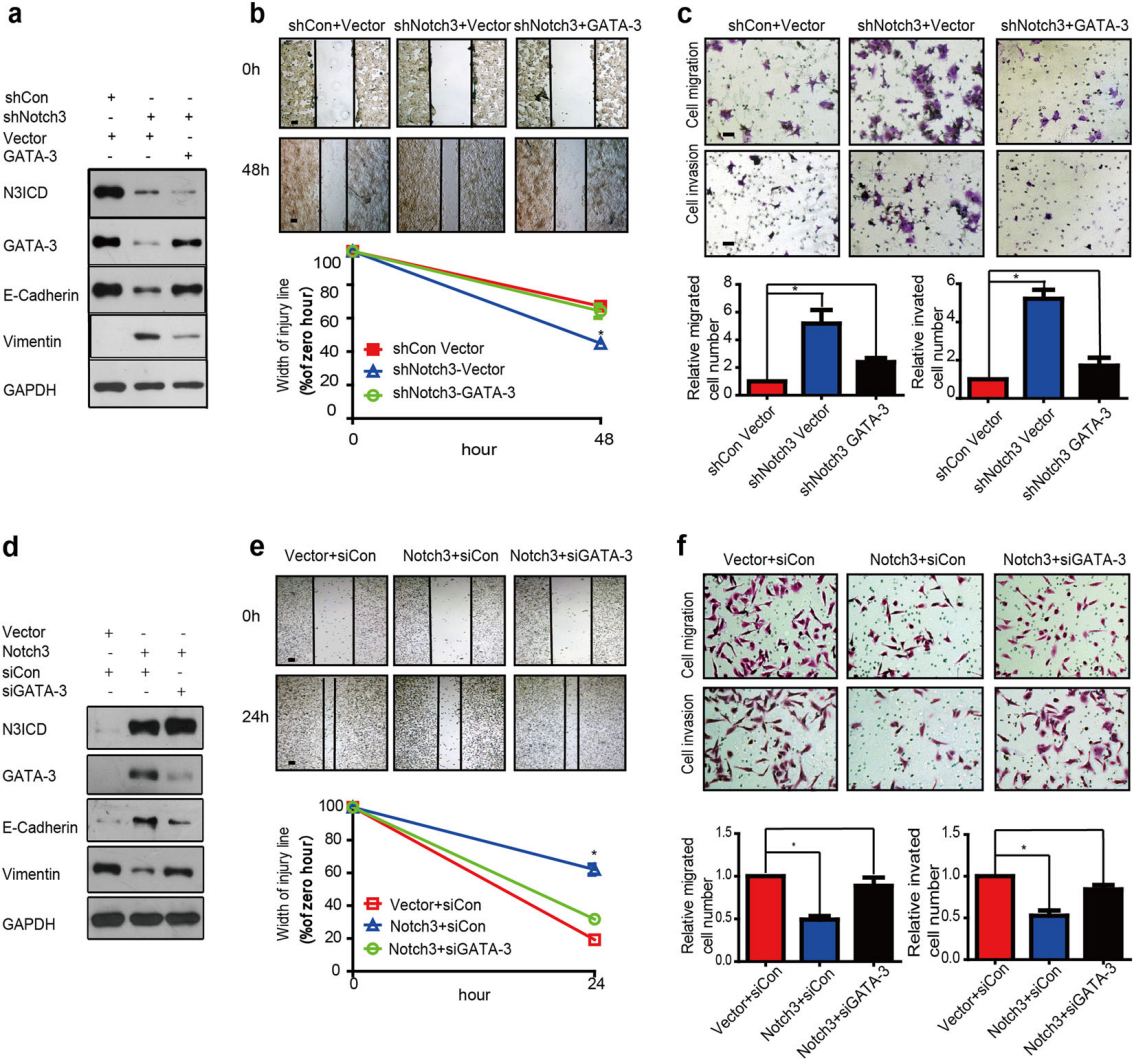
Notch3 knockdown in MCF-7 cells promotes EMT while N3ICD overexpression in MDA-MB-231 cells inhibits EMT. **a** Expression of Notch3, GATA-3, E-cadherin, and vimentin in MCF-7 cells analyzed by western blot when stably silencing Notch3, with or without overexpression of GATA-3. **b** Wound healing assay showed the knockdown of Notch3 enhanced the cellular motility of MCF-7 cells; such changes could be rescued by simultaneously adding a GATA-3 plasmid. Representative pictures (top) and quantitative data (bottom) of wound recovery after 48 h cell culture. Each sample contained three wells. **c** Representative pictures (top) and quantitative data (bottom) of migration or invasion assays. Each sample contained three wells. **d** Expression of Notch3, GATA-3, E-cadherin, and vimentin in MDA-MB-231 cells analyzed by western blot when stably overexpressing N3ICD, with or without knockdown of GATA-3 by siRNA. **e** Representative pictures (top) and quantitative data (bottom) of wound recovery after 24 h cell culture. Each sample contained three wells. **f** Representative pictures (top) and quantitative data (bottom) of migration or invasion assays. Each sample contained three wells. All experiments were performed at least three times and data were statistically analyzed by two-sided *t*-test. *P < 0.05, **P < 0.01, ***P < 0.001 vs. control. Error bars indicate standard error of the mean (SEM)

Due to the important roles of Notch3 and GATA-3 as epithelial phenotype markers of breast cancer, we hypothesized that the Notch3/GATA-3 axis modulates the expression of EMT markers. In an attempt to evaluate the effect of the Notch3/GATA-3 axis in regulating EMT, we examined EMT marker expression after knocking down Notch3 in MCF-7 cells with/without enforced expression of GATA-3. When knocking down Notch3 in MCF-7 cells, epithelial marker E-cadherin expression decreased, while that of vimentin, a mesenchymal marker, increased as compared to controls (Fig. 4a). We next assessed EMT-associated changes in cell morphology when Notch3 was stably knocked down in MCF-7 cells. While control cells maintained strong cell contacts and epithelial morphology, a subset of cells expressing Notch3/GATA-3 shRNA presented with a fibroblastic morphology, demonstrating scattering characteristic of a loss of cell–cell adhesion (Supplementary Fig. S1c). We next assess influence of Notch3 knockdown on the motility breast cancer cell via wound healing assay. After culturing for 48 h, the width of the scratch wound in shNotch3 MCF-7 cells had reduced to only 44% as compared to 0 h, while the width of the scratch wound in control cells had reduced to 64% as compared to 0 h, indicating that loss of Notch3 expression promotes cell motility. The gap was restored to 55% by enforced GATA-3 expression, suggesting that reduced cell motility induced by loss of Notch3 be mediated by GATA-3 (Fig. 4b). We further assessed the role of Notch3 in MCF-7 cells via migration assay. Suppression of Notch3 expression via shRNA increased cell migration by 5-fold, which was partially rescued by overexpressing GATA-3. Similar results were also found in in vitro invasion assays (Fig. 4c). These findings indicate that loss of Notch3 expression in MCF-7 cells leads to increased migratory and invasive capacities by downregulating GATA-3.

In contrast, the overexpression of N3ICD in MDA-MB-231 cells led to decreased expression of vimentin and increased expression of E-cadherin, and these changes were reversed by knocking down GATA-3 (Fig. 4d). Regarding morphological analysis, MDA-MB-231 control cells appeared more mesenchymal, typically appearing spindle-shaped. However, when stably transfected with N3ICD/GATA-3, MDA-MB-231 cells appeared more epithelial in shape (Supplementary Fig. S1d). In the wound healing assay, the migration of MDA-MB-231 control cells demonstrated as independent single cells, whereas those expressing N3ICD migrated as a sheet, maintaining cell–cell contacts (Fig. 4e). Furthermore, after culturing for 24 h, the scratch wound width in MDA-MB-231-N3ICD cells had healed to 62% as compared to 0 h, dwarfing the 19% gap healed by MDA-MB-231 control cells. This result suggests that overexpression of Notch3 was capable for suppressing the migratory capability of MDA-MB-231 cells. However, the gap had healed to only 31% when GATA-3 expression was reduced with siRNA (Fig. 4e). Migration assays revealed that fewer MDA-MB-231-N3ICD cells successfully migrated (~30.4%) than cells transfected with control vector after culture for 48 h. Nonetheless, GATA-3 knockdown via siRNA increased the number of migrating cells by over two-fold as compared to N3ICD-expressing cells. We observed similar results via cell invasion experiment (Fig. 4f). These data indicate that Notch3 induces GATA-3 expression to inhibit migratory and invasive capabilities in MDA-MB-231 cells.

### Notch3 inhibits metastatic capacity of breast cancer in vivo model by regulating GATA-3

**Fig. 5:**
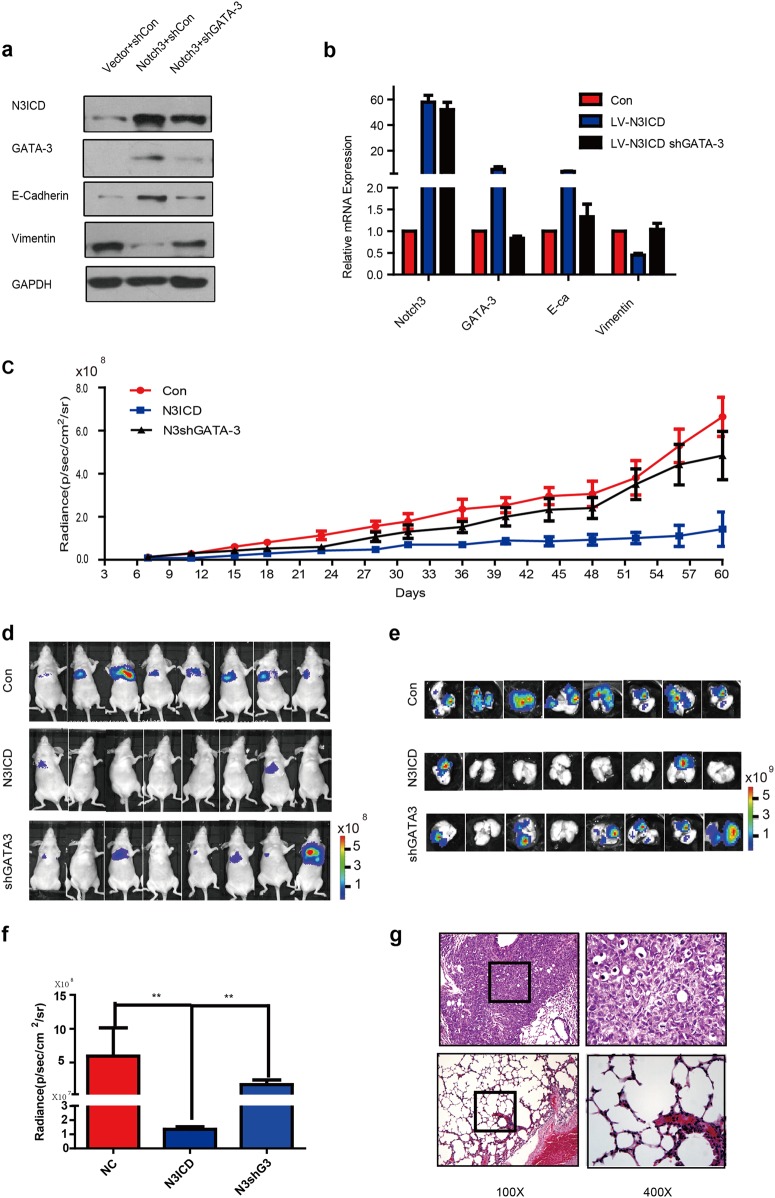
Notch3 inhibits distant metastasis via regulation of GATA-3 in a humanized mouse model. **a**–**b** Expression of Notch3, GATA-3, E-cadherin, and vimentin in MDA-MB-231 cells analyzed by western blot (**a**) or RT–PCR (**b**) when stably overexpressing N3ICD, with or without knockdown of GATA-3 by lentivirus infection. **c**–**d** NU/NU mice were injected intravenously with MDA-MB-231 cells (1 × 10^6^ cells) for the control group, Notch3 stably expressing group, or the stably expressing Notch3 with stably knocked down GATA-3 group. Bioluminescence imaging was performed 1 h after injection and then twice a week (**c**). The photon intensities of lung metastases in vivo in the three groups are indicated (mean ± SEM) (**d**). **e**–**f** Mice were sacrificed to examine metastases 2 months after injection. Representative bioluminescence imaging was performed and mice were euthanized immediately after imaging (*n* = 8 independent mice per group). Bioluminescence imaging of a mouse lung which was taken out within 5 min of sacrifice (**e**). The photon intensities of lungs in vitro in the three groups are indicated (**f**) (mean ± SEM). **g** Representative hematoxylin and eosin (HE) staining for lung metastases in the two groups (Magnification: ×100 and ×400

We used MDA-MB-231-N3ICD cells to investigate the function of the Notch3/GATA-3 axis in distant spreading of breast cancer, with/without GATA-3 knockdown, in tumor xenograft models. Confocal fluorescence microscopy confirmed successful construction of MDA-MB-231 cells stably overexpressing N3ICD, with or without GATA-3 knockdown by lentivirus infection (Supplementary Fig. S2a; Fig. 5a–b). The photon intensities of different cell numbers in the three groups showed that the bioluminescence image system can be used to observed distant metastases in vivo (Supplementary Fig. S2b–c). MDA-MB-231 cells were injected into in vivo mouse models via tail veins to determine the effect of Notch3 on metastasis (Fig. 5c). Mouse body weight and organ bioluminescence imaging (BLI) were observed and recorded (Supplementary Fig. S2d; Fig. 5d). Sixty days after injection, the mice were humanely euthanized, and various organs were sampled to enumerate the number of metastases via BLI analysis. We detected lung metastases in all mice that were injected with control vector-transfected MDA-MB-231 cells (*n* = 8). Only two mice injected with N3ICD-overexpressing cells had observable metastases (*n* = 8; Fig. 5e–f). We validated that overexpression of Notch3 inhibited lung metastasis and suppressed vessel invasion using ex vivo BLI imaging and hematoxylin–eosin (H&E) staining (Fig. 5g–h). Breast cancer metastases were not found in livers, spleen, stomach, brain, or kidneys from all three groups (Supplementary Fig. S2e). These results imply that Notch3 is capable of inhibiting the spreading of breast cancer in vivo.

### Expression of Notch3 and GATA-3 correlate with ERα positivity in breast cancer patients

**Fig. 6:**
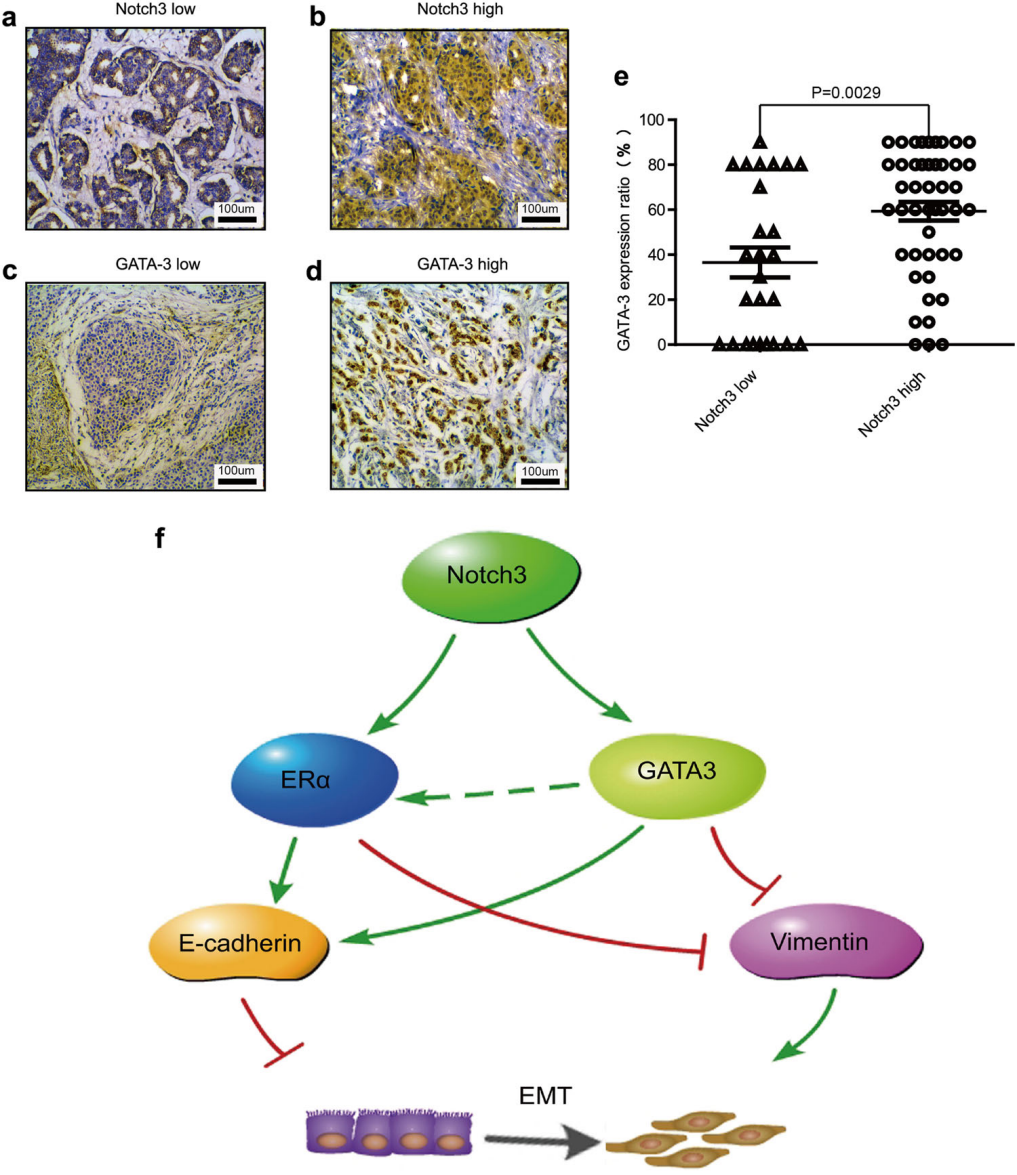
Notch3 expression is associated with GATA-3 in breast cancer clinical cases. **a**–**d** Representative pictures of Notch3 low expression (**a**), Notch3 high expression (**b**), GATA-3 low expression (**c**), and GATA-3 high expression (**d**). Samples were stained by immunohistochemistry. **e** Positive correlation between Notch3 and GATA-3 expression (*P* = 0.0029) analyzed by two-sided *t*-test. *P < 0.05, **P < 0.01, ***P < 0.001 vs. control. **f** Proposed model of how Notch3 and GATA-3 regulate EMT in breast cancer

Our previous work reported a high correlation between Notch3 and ER expression profiles across different molecular subtypes of breast cancer^29^. In addition, evidence from a public database consistently indicated that GATA-3 expression is significantly associated with ER expression in breast cancer^30^. We therefore determined expression of Notch3 and GATA-3 in tissue samples from breast cancer patients with immunohistochemistry (IHC) (Fig. 6a–d) and explored their possible relationship with progesterone receptor (PR) and ER statuses, as well as their associations with other clinicopathologic features. A total of 72 patients enrolled the study consecutively from 2007 to 2008 with breast cancer diagnosed and consequently received surgery at the Breast Center, Cancer Hospital of SUMC. Notch3 localized in both the nuclei and the cytoplasm according to IHC analysis (Fig. 6b), while GATA-3 was exclusively localized to the nucleus (Fig. 6d). Of the 72 cases with IHC analysis, 43 cases were positive in both nucleus and cytoplasm, 1 case with only cytoplasmic expression, and 2 cases with exclusive nuclear expression (the expression pattern of Notch3 showed in Supplementary FigureS4), while 26 breast cancer cases were negative for expression of Notch3. Notch3 expression either in nuclear or in cytoplama was strongly associated with that of GATA-3 in breast cancer tissue samples (Fig. 6e, Table 1; Pearson *r* = 0.408, *P* = 0.001). Of all patients, 48 were ERα positive (48/72; 66.7%), and 24 were ERα negative (24/72; 33.3%), respectively. Compared to ERα negative tumors, levels of Notch3 and GATA-3 were significantly higher in ERα positive tumors (Table 2; *P* = 0.027, and 0.038, respectively). Likewise, Notch3 and GATA-3 expression levels were statistically higher in PR positive than in PR negative tumors (Table; *P* = 0.048, and = 0.027, respectively). Moreover, in the analysis of lymph node status, we found that Notch3/GATA-3 positive patients exhibited fewer lymph node metastases than Notch3/GATA-3 negative patients (Table 2; *P* = 0.026, *P* = 0.021). All data indicate that Notch3 expression positively correlates with elevated expression of GATA-3, ER and PR, as well as with a lower risk of lymph node metastasis. Additional clinicopathologic variables are showed in Table 2 and Supplementary Table S3. Representative pictures of different Notch3 or GATA-3 expression levels are showed in Supplementary Figure S5.

**Table 1:** Correlation of Notch3 and GATA-3 expression in patients with breast cancer

Notch3	GATA-3	**−**	**+**	*X* ^2^	*r*	*P*-value
**−**		14	15	11.975	0.408	=0.001^*^
**+**		5	38			

^*^P-value <0.05 was considered statistically significant

**Table 2:** Correlation of Notch3 or GATA-3 expression with clinicopathological status in 72 patients with breast cancer

Clinicopathologic features	Notch3^-^(*n* = 29)	Notch3^+^ (*n* = 43)	*P*	GATA-3^-^ (*n* = 19)	GATA-3^+^ (*n* = 53)	*P*
Age at diagnosis			0.598			0.603
<50	13(44.8%)	22(51.2%)		8(42.1%)	26(49.1%)	
≥50	16(55.1%)	21(48.8%)		11(57.9%)	27(50.9%)	
Menstrual conditions			0.35			0.303
premenopausal	15(51.7%)	27(62.8%)		9(47.4%)	29(54.7%)	
postmenopausal	14(48.3%)	16(37.2%)		10(52.6%)	24(45.3%)	
Tumor size (cm)			0.095			0.109
<2	12(41.3%)	8(18.6%)		7(36.8%)	13(24.5%)	
≥2, <5	11(37.9%)	25(58.1%)		11(57.9%)	25(47.2%)	
≥5	6(20.7%)	10(23.3%)		1(5.7%)	15(28.3%)	
LN metastasis			0.026^*^			0.021^*^
0	13(44.8%)	29(67.4%)		9(47.4%)	33(62.3%)	
1–3	2(6.9%)	6(14.0%)		1(5.7%)	11(20.8%)	
≥4	14(48.3%)	8(18.6%)		9(47.4%)	9(17.0%)	
Histological grade			0.287			0.963
I	3(10.3%)	9(20.9%)		3(15.8%)	9(17.0%)	
II	15(51.7%)	24(55.8%)		10(52.6%)	29(54.7%)	
III	11(37.9%)	10(23.6%)		6(31.6%)	15(28.3%)	
Stage			0.411			0.111
I–II	17(58.6%)	29(67.4%)		15(78.9%)	31(58.5%)	
III–IV	12(41.3%)	14(32.6%)		4(21.1%)	22(41.5%)	
ER			0.027^*^			0.038^*^
Positive	15(51.7%)	33(76.7%)		9(47.4%)	39(73.6%)	
Negative	14(48.3%)	10(23.6%)		10(52.6%)	14(26.4%)	
PR			0.048^*^			0.027^*^
Positive	13(44.8%)	29(67.4%)		7(36.8%)	35(66.0%)	
Negative	16(55.2%)	14(32.6%)		12(63.2%)	18(34.0%)	
HER-2			0.552			0.64
Positive	10(34.5%)	12(27.9%)		5(26.3%)	17(32.1%)	
Negative	19(65.5%)	31(72.1%)		14(73.7%)	36(67.9%)	
Ki67 status			0.814			0.128
<14%	17(58.6%)	24(55.8%)		8(42.1%)	33(62.3%)	
≥14%	12(41.4%)	19 (44.2%)		11(57.9%)	20(37.7%)	
Breast cancer subtypes			0.169			0.28
Luminal A	9(31.0%)	25(58.1%)		7(36.8%)	27(50.9%)	
Luminal B/HER-2 Neg	4(13.8%)	4(9.3%)		1 (5.7%)	7(13.2%)	
Luminal B/HER-2 Pos	2(6.9%)	4(9.3%)		1 (5.7%)	5(9.4%)	
HER-2	5(17.2%)	4(9.3%)		3(15.8%)	6(11.3%)	
TNBC	9(31.0%)	6(14.0%)		7(36.8%)	8(15.1%)	

*LN* lymph node, *ER* estrogen receptor, *PR* progesterone receptor, *HER-2* human epidermal growth factor receptor-2

^*^P-value < 0.05 was considered statistically significant

## Discussion

This study represents the first demonstration that Notch3 transcriptionally upregulates the expression of GATA-3 and its downstream genes by a direct binding to the CSL core elements within its promoter, leading to repression of EMT in breast cancer cells, as well as suppression of in vivo distant metastasis. Of all the GATA family factors, GATA-3 is considered an emerging and specific biomarker of breast cancer that shows less invasive, has fewer metastases, and therefore associates with a better prognosis. ERα-positive, luminal subtype, or well-differentiated epithelial breast cancer expresses a notably high level of GATA-3 compared to ER-negative invasive breast cancer^31,32^. GATA-3, known to be abundantly expressed in luminal cells, plays a crucial role during epithelial proliferation and is required for development and formation of normal mammary gland^6,7^. Thus, GATA-3 is believed to maintain a luminal phenotype by upregulating ERα. Solid evidence exists showing that, in breast cancer, the expression of GATA-3 is highly correlated to that of ERα, and the results were consistent with our previous findings^33^.

Our investigation of the regulatory mechanism of Notch3 and GATA-3 found that both were positively associated with ER expression in breast cancers. Interestingly, enforced N3ICD expression results in the upregulation of GATA-3 in breast cancer cell lines with ERα-negative phenotype, while suppressing Notch3 expression downregulates GATA-3 expression in ERα-positive cells. Moreover, using a reporter assay and a ChIP assay, our study demonstrates that Notch3 is capable of binding to CSL-binding motifs in the promoter of GATA-3 in breast cancer cells, suggesting that N3ICD directly activates GATA-3 expression. To date, there have been few investigations into the specific connection between Notch3 and GATA-3 in breast cancer cells. GATA-3 is a critical transcription factor that controls early development and formation of embryonic mammary gland and differentiation of T lymphocyte in response. Several studies have previously demonstrated that GATA-3 was a direct Notch1 downstream target through RBP-J, acting cooperatively with Notch signaling to generate optimal Th2 cell responses^34^. Studies also revealed that the canonical Notch pathway and GATA-3 are both required by differentiated luminal cells to maintain a quiescent state^35^. However, during the commitment to mammary epithelial cells or tumorigenesis of breast cancer cells, whether GATA-3 is the direct target of certain specific Notch receptor has not been investigated^19^.

As previously reported, in recent studies we found that EMT of ERα-negative subtype breast cancer cells inhibits by overexpressing N3ICD, while drastically promoted by Notch3 knocking down^29^. In this study, ectopic GATA-3 expression partially rescued Notch3 silencing-induced EMT transition, while GATA-3 knockdown reversed Notch3^−^ induced mesenchymal–epithelial transition (MET). Thus, these results strongly indicate and support that Notch3, at least in part, acts via GATA-3 induction to inhibit EMT in breast cancer cells. These observations are supported by the finding that GATA-3 may act as a luminal epithelial biomarker of breast cancer. For example, Si et al. reported that GATA-3 inhibits the expression of genes having been recognized to be involved in EMT, such as ZEB2, TGFB1, MDM2, ZNF217, and BCS3, by recruiting the complex of G9A/NuRD (MTA3)^36^. Regarding the role of GATA-3 during the EMT process, Yan et al. also demonstrated that GATA-3 can activate the E-cadherin promotor by binding to typical GATA-like motifs, thereby reversing EMT^10^. Ohashi et al. demonstrated that Notch3 is necessary to limit the expression of ZEBs, which are transcription factors that play critical roles in TGF-beta–mediated EMT, thus describing the mechanism for Notch signaling pathway in the progression of esophageal squamous cancers^37^. Together, these findings suggest a functional and positive correlation between Notch3 and GATA-3 during EMT in breast cancer. However, given the emerging evidence that Notch receptors have distinct activities and functions in different tissues, Notch3 may have a unique role in breast cancer cells^19^. Interestingly, a prior study has described a positive feedback loop regulating between expression of GATA-3 and ERα in breast cancer^6,38^. Taken together, Notch3 may inhibit EMT through a multi-tiered system such as directly or indirectly upregulating ERα (Fig. 6f). Wang et al. provided evidences that Notch3 play a pivotal role in the cellular response to hypoxia in malignant cells, i.e., hypoxia initially stabilizes HIF1α, and subsequently, upregulates Notch3, which in turn decreases the expression of IL6. In our unpublished data, there are negative correlations between Notch3/GATA-3 and IL6 at both protein and mRNA levels, suggesting Notch3/GATA3 may impact on metastases by altering microenviroment, i.e., IL-6 induction (shown in the Supplementary Table S4). However, the exact mechanisms whereby Notch3–GATA-3 or Notch3–ERα function in microenvironmental- or condition-specific manners require further investigation.

Notably, Chou et al. reported that GATA-3 induces miR-29b to facilitate differentiation, repress metastasis and remodel the microenvironment in breast cancer^39^. Si et al. reported that disrupting the feedback loop between GATA-3 and ZEB2-induced repression programs promotes EMT and contributes to distant metastasis of breast cancer^36^. Using tumor xenograft models, we demonstrate herein that Notch3 restrains metastasis of breast cancer in vivo via regulation of GATA-3. Although the relationship between EMT and distant metastasis remains controversial; plenty of evidence points to the high correlation between EMT and distant metastases in breast cancer, including the latest study by Ye Xin et al.^40^. Considering that dysfunction of GATA-3 may correlate to distant metastases by reversing EMT, in combination with the observation that Notch3 positively regulates GATA-3 expression in vitro, we strongly proposed that Notch3 may be an essential factor in suppressing metastasis in breast cancer in some measure by inducing GATA-3 and inhibiting EMT.

The analysis of clinical data further confirmed the co-expression and correlation of three transcriptional factors, i.e., Notch3, GATA-3, and ERα. These findings are supported by Hisamatsu et al. who report that GATA-3 is connected to a less aggressive, HR-positive, HER2-negative phenotype, and therefore with a favorable survival outcome in breast cancer patients^30^. Moreover, we found that Notch3 and/or GATA-3 negative patients exhibited more lymph node metastases; this implies that the absence or low expression of Notch3/GATA-3 may indicate a high potential for metastasis. Yoon et al. reported that higher GATA-3 levels are indicator of favorable survival in breast cancer patients^12^. Our previous work demonstrated that the high expression of Notch3 protein or mRNA predicted better relapse-free survival (RFS) in ER-positive breast cancer patients^29^. Given that both Notch3 and GATA-3 suppress EMT, the above findings are relevant in improving the former’s prognostic significance in patients with breast cancer. Altogether, we have presented evidence dissecting the role of the Notch3/GATA-3 axis in the EMT process, as well as in the inhibition of breast cancer metastasis. In addition, we further describe a likely molecular mechanism controlling this axis. Our results provide novel insights into the complex regulation of EMT and provide a basis for further delineation of the Notch3/GATA-3 pathway as a promising candidate of prognostic indicator and/or therapeutic avenue for breast cancers.

## Materials and methods

### Cell culture and transfection

All cell lines, including MCF-7, T47D, SK-BR3, MDA-MB-231, and BT549, were obtained from the American Type Tissue Collection (ATCC) and cultured followed manufacturer’s instructions. For Notch3 or GATA-3 knockdown in MCF-7 cells, specific siRNAs targeting Notch3 and GATA-3, as well as control siRNAs were designed and synthesized by GenePharma (Suzhou, China). Oligonucleotide siRNAs are listed (see Supplementary Table 3). pCLE-N3ICD (Plasmid 26894) and control vector pCLE (Plasmid 17703) were purchased from Addgene (Cambridge, MA, USA). The plasmid, pMIG hGATA-3, and its control vector, pMIG, were gifts from Professor Zena Werb (Department of Anatomy, University of California, San Francisco, USA).

A stable Notch3-silenced cell line, MCF-7shN3, was generated by stable transfection with a silencing vector, pGPU6/GFP/Neo-shNotch3#1, containing an oligonucleotide sequence targeting Notch3 or a scramble sequence in the control vector (Supplementary Table S1, GenePharma). A stable Notch3 ICD overexpressing cell line, MDA-MB-231-N3ICD, was generated by stably transfecting parental MDA-MB-231 cells with the pCLE-N3ICD vector. For both shRNA and DNA plasmid transfection assays, Lipo2000 (Life Technologies, Carlsbad, CA, USA) was used according to the manufacturer’s instructions. For the selection of transfectants, 1 μg/mL G418 was added to the medium after 2 days in culture.

### Reverse transcription and PCR analysis

Total RNA purification, reverse transcription and real-time PCR analysis were performed as described in our previous works^29^. To normalize the amount of mRNA in each sample, β-actin was used. Primer used in PCR assay were showed in Supplementary Table S1.

### Western blotting analysis

Western blotting was processed used methods we described previously^41^. Antibodies used and volume dilution were listed in Supplementary Table S2.

### Immunohistochemistry

Samples of breast cancer tissues processing and immunohistochemistry staining as well as pathological scoring were performed as previously described^29^. Pathological scoring was investigated by two independent observers (X-L.W., H-Y.L.). Antibodies used in IHC staining are listed in Supplementary Table S2.

### Wound healing assay

Cells were cultured in medium with Mitomycin C at a concentration of 25 mg/mL for 30 min. Then, injury lines were applied. When cells were culture at 90% confluency, injury line was made using a 2-mm wide tip on the culture plates. After rinsing with phosphate-buffered saline (PBS), cells were cultured with complete medium and allowed to migrate, and photographs were taken (×40) after 24 h (MDA-MB-231) or 48 h (MCF-7). Five random widths were recorded and measured for quantitation in each injury line.

### Transwell invasion assays

Cell culture inserts (8 μM pore size; BD, Franklin Lakes, NJ, USA) and Matrigel invasion chambers (BD) were used to perform migration and invasion assays, respectively. After serum-starved for 24 h, 2 × 10^4^ MDA-MB-231 cells or 5 × 10^4^ MCF-7 cells were seeded into the upper chamber cultured in serum-free medium, while the bottom chamber was added with complete medium. For migration assays, cells were stained with 0.1% crystal violet after 24 h. For invasion assays, the cells were stained with 0.1% crystal violet after 48 h. Each experiment was carried out in triplicate. The exact number of cells from 5 random fields in every individual well was captured and calculated by two investigators (H-Y.L., Y-K.L.).

### Immunofluorescence

Immunofluorescence of MCF-7, T47D, and knocked down Notch3 MCF-7siN3 or T47DsiN3 was processed used followed instructions of manufacturer’s as we reported previously^41^. Antibodies used and volume dilution were listed in Supplementary Table S2. Slides were checked under a Zeiss microscope (Zeiss, Oberkochen, Germany).

### Lentiviral and retroviral production

Viral production was carried out using calcium-phosphate–mediated transfection of 293 T cells. Virus was concentrated by ultracentrifugation, and added to cells with polybrene transfection agent. Stably transduced cells were constructed and selected through cultured in a variety of medium with G418, puromycin, or hygromycin for at least 5 days, or gathered by a fluorescence-activated cell sorting system.

### Transient transfection experiments and luciferase reporter assays

The GATA-3 promoter region (−1000 upstream of exon 1 and extending to −1 bp) was cloned into the HandIII/XhoI sites of the luciferase reporter vector, pGL3-basic (Panomics, Fremont, CA, USA), named GATA-3 Wt luc. The mutant type of the GATA-3 promoter, named GATA-3 Mt luc, was created in the same reporter vector with a deletion of the CSL-binding site 1 (−829 to −834 bp upstream of exon 1) and CSL-binding site 2 (−665 to −670 bp upstream of exon 1). Transfection were performed using Lipo2000 based on the manufacturer’s instructions. The control vector, pRL-SV40 (Promega, Fitchburg, WI, USA) was utilized to balance the efficiency of transfection. To determine the influence of silenced or overexpressed Notch3 on GATA-3 Wt luc activities, GATA-3 Mt luc was simultaneously co-transfected into MCF-7 or MDA-MB-231 cells with various doses of siRNA or overexpression vectors. Luciferase activity was determined using a dual luciferase reporter assay kit (Promega).

### Chromatin immunoprecipitation assays

ChIP assays was performed as previously described^29^, when MCF-7 cells cultured in a 100-mm dish were in 80% confluence. Cell lysates incubated with IgG (Santa Cruz Biotechnology) were taken as an IP control, or incubated with an antibody specific to Notch3 (CST) as experimental group. Input PCR amplification was carried out with 10% of total cell lysates. The PCR reaction amplified 165 bp (located −969 to −804 bp, region 1) and 166 bp products (located −781 to −665 bp, region 2) from the GATA-3 promoter that contained the CSL-binding elements (GGGAA, located −829 to −825 bp and −665 to −661 bp upstream of exon 1). A 354 bp PCR product (located −969 to −665 bp region 3) containing both two binding elements was also amplified for ChIP assay, and a 150 bp PCR reaction product (located −628 to −386 bp region 4), which was close to the CSL-binding elements but did not contain the sequence, was utilized as negative control. Sequences of primers and all the antibodies used in ChIP assays are showed in Supplementary Tables S2 and S3, respectively.

### Electrophoretic mobility shift assay

Nuclear extracts obtained from MCF-7 cells were used in the EMSA experiments. Oligonucleotides that contained the core sequence of CSL-binding elements were used in the assay and performed according to manufacturer’s protocols (Viagene, Tampa, Florida, USA). In competition assays, excessive amounts of unlabeled competitors were added 20 min before the addition of labeled probes. For supershift assays, an anti-Notch3 monoclonal antibody (5 μg, CST) was added and incubated at 4 °C for 60 min. Sequences of the probes and mutated competitors in the EMSAs are displayed in Supplementary Table S1.

### Tumor xenograft models

All animal protocols were strictly followed the guidance issued by the Institutional Animal Care and Use Committee of Shantou University Medical College (SUMC). 6-week-old female Nu/Nu nude mice (purchased from Vital River Laboratories, Beijing, China) were used for all the animal experiments. In brief, MDA-MB-231-NC, MDA-MB-231-N3ICD, or MDA-MB-231-N3ICD/shGATA-3 cells were injected randomly via the lateral tail vein (1 × 10^6^ cells). The mice growth was monitored by measuring body weights twice a week. The development of distant organ metastasis was monitored and recorded by an IVIS Kinetic Imaging System (PerkinElmer, Waltham, MA, USA) in every three days. Before 15 min of imaging, the mice were injected intraperitoneally with 150 mg/kg D-luciferin (PerkinElmer). Vein tail injection mice were sacrificed after 31 days. At the time of killing, organs were harvested, with subsequent visualization by an IVIS Kinetic Imaging System. Lungs, brain, spleen, kidneys, stomach, and liver were excised, and then fixed in 4% paraformaldehyde with final step of paraffin embedding. H&E staining was performed for tumor phenotype recognition. Tumor formations were identified by two pathologists of the Department of Pathology in Cancer Hospital of SUMC.

### Statistical analysis

All experiments were performed in triplicate. Data are shown as the mean ± standard error of the mean (SEM). A two-sided Student’s *t*-test were used for the statistical analysis. *P*-value of less than 0.05 was considered statistically significant.

### Study approval

The study protocol was reviewed and approved by the ethics committee of the Cancer Hospital of Shantou University Medical College. All patients enrolled in this study have signed written informed consents. The animal experiment protocol was approved by the Institutional Animal Care and Use Committee of SUMC.

## Electronic supplementary material

Supplementary
