# Supplementary material for: Notch3 inhibits epithelial–mesenchymal transition in breast cancer via a novel mechanism, upregulation of GATA-3 expression
Source: Oncogenesis. 2018 Aug 13;7(8):59. doi: 10.1038/s41389-018-0069-z (PMC6087713; doi:10.1038/s41389-018-0069-z)
Supplement: Supplementary file 1 — Supplementary [file 41389_2018_69_MOESM1_ESM.doc]

**Supplementary**

**Table S1. Oligonucleotide sequences for siRNA constructs used in real-time PCR and CHIP and EMSA assays**

| **Assay** |  | **Sequences (5' to 3')** | **Amplicon (bp)** |  |
| --- | --- | --- | --- | --- |
| RT-PCR |  |  | |  |
|  | Notch3 | F ATGCAGGATAGCAAGGAGGA | 86 |  |
|  |  | R AAGTGGTCCAACAGCAGCTT |  |  |
|  | GATA-3 | F AGCCACTCCTACATGGACGC | 98 |  |
|  |  | R AAGGGGCTGAGATTCCAGGG |  |  |
|  | ERα | F TGCTTCAGGCTACCATTATGGA | 98 |  |
|  |  | R TGGCTGGACACATATAGTCGTT |  |  |
|  | E-cadherin | F AAAGGCCCATTTCCTAAAAACCT | 172 |  |
|  |  | R TGCGTTCTCTATCCAGAGGCT |  |  |
|  | Vimentin | F GACGCCATCAACACCGAGTT | 238 |  |
|  |  | R CTTTGTCGTTGGTTAGCTGGT |  |  |
|  | β-actin | F AGCGAGCATCCCCCAAAGTT | 264 |  |
|  |  | R GGGCACGAAGGCTCATCATT |  |  |
|  | GAPDH | F ATGGGGAAGGTGAAGGTCG | 108 |  |
|  |  | R GGGGTCATTGATGGCAACAATA |  |  |
|  | Hes1 | F AGTGAAGCACCTCCGGAAC | 107 |  |
|  |  | R CGTTCATGCACTCGCTGA |  |  |
|  | Hey1 | F CATACGGCAGGAGGGAAAG | 125 |  |
|  |  | R GCATCTAGTCCTTCAATGATGCT |  |  |
| siRNA |  |  |  |  |
|  | siNotch3 #1 | CUCCUCCUUGCUAUCCUGCAUGUCCUU |  |  |
|  | siNotch3 #2 | UAUAGGUGUUGACGCCAUCCACGCA |  |  |
|  | siNotch3 #3 | GAGCCAAUAAGGACAUGCA |  |  |
|  | siGATA-3#1 | AAGCCUAAACGCGAUGGAUAU |  |  |
|  | siGATA-3#2 | AACAUC-GACGGUCAAGGCAAC |  |  |
|  | siGATA-3#3 | TGCCTGTGGGCTCTACTAC |  |  |
|  | siNC | UUCUCCGAACGUGUCACGU |  |  |
| ChIP |  |  |  |  |
|  | Region1 | F GAGGGCTGGTTTCCTTGACT | 165 |  |
|  |  | R GGAGGAAGAGACTGGCTCTA |  |  |
|  | Region2 | F TGTTGCCACTCAAGTCAAAAGC | 166 |  |
|  |  | R ATGCCTTTGACTGGAGCGTC |  |  |
|  | Region3 | F GAGGGCTGGTTTCCTTGACT | 354 |  |
|  |  | R ATGCCTTTGACTGGAGCGTC |  |  |
|  | Region4 | F CCTTTATCCGATGACTCACC | 150 |  |
|  |  | R AGGATCTAATGCAGGGTGTT |  |  |
| EMSA |  |  |  |  |
|  | Probe1 or competitor 1 (P1) | GCGTACTCG**GGGAA**TGAGTTAG |  |  |
|  | P1 mutant | GCGTACTCG**TGTGA**TGAGTTAG |  |  |
|  | Probe2 or competitor 2 (P2) | CAGAAGGCTC**GGGAA**AGAGGTGA |  |  |
|  | P2 mutant | CAGAAGGCTC**ATAGC**AGAGGTGA |  |  |

**Table S2. Proteins and description of corresponding antibodies**

| **Antibodies** | **Vendor** | **Source** | **KDa** | **Dilution** |
| --- | --- | --- | --- | --- |
| Notch3 | CST/ D11B8 | Rabbit/mono | 90 | 1:3000(WB)、1:200(IF) |
| Notch3 | Santa Cruz/M-20 | Goat/poly | 90 | 1:200 (IHC) |
| GATA-3 | CST/D13C9 | Rabbit/mono | 50 | 1:3000（WB） |
| GATA-3 | Santa cruz/HG3-35 | Mouse/mono | 50 | 1:100(IF) 1:200(IHC) |
| ERα | CST /D6R2W | Rabbit/mono | 68 | 1:3000 (WB) |
| E-cadherin | DAKO/NCH-38 | Rabbit/mono | 120 | 1:3000 (WB) |
| Vimentin | CST/D21H3 | Rabbit/mono | 55 | 1:2000 (WB) |
| GAPDH  β-actin | Santa Cruz/sc-32233  Santa Cruz/[sc-58673](https://www.scbt.com/scbt/product/actin-antibody-2q1055?requestFrom=search) | Mouse/mono  Mouse/mono | 37  42 | 1:3000 (WB)  1:3000 (WB) |

**Table S3:** Correlation of Notch3 or GATA-3 Expression with Clinicopathological Status in 72 Patients with Breast Cancer

| Clinicopathologic features | GATA-3^-^&Notch3^-^ (n=14) | GATA-3^+^&Notch3^+^  (n=37) | *P* | GATA-3^-^&Notch3^+^(n=15) | GATA-3^+^&Notch3-(n=6) | *P* |
| --- | --- | --- | --- | --- | --- | --- |
| Age at diagnosis |  |  | 0.407 |  |  | 0.577 |
| <50 | 5(35.7%) | 18(48.6%) |  | 8(53.3%) | 4(66.7%) |  |
| ≥50 | 9(64.3%) | 19(51.4%) |  | 7(46.7%) | 2(33.3%) |  |
| Menstrual conditions |  |  | 0.431 |  |  | 0.577 |
| premenopausal | 7(50.0%) | 23(62.2%) |  | 8(53.3%) | 4(66.7%) |  |
| [postmenopausal](javascript:void(0);) | 7(50.0%) | 14(37.8%) |  | 7(46.7%) | 2(33.3%) |  |
| Tumor size (cm) |  |  | 0.091 |  |  | 0.022* |
| <2 | 7(50.0%) | 8(21.6%) |  | 5(33.3%) | 0(0.0%) |  |
| ≥2，<5 | 6(42.9%) | 19(51.3%) |  | 5(33.3%) | 6(100.0%) |  |
| ≥5 | 1(7.1%) | 10(27.0%) |  | 5(33.3%) | 0(0.0%) |  |
| LN metastasis |  |  | 0.028* |  |  | 0.423 |
| 0 | 5(35.7%) | 24(64.8%) |  | 8(53.3%) | 5(83.3%) |  |
| 1–3 | 1(7.1%) | 6(16.2%) |  | 1(6.7%) | 0 (0.0%) |  |
| ≥4 | 8(57.1%) | 7(18.9%) |  | 6(40.0%) | 1(16.7%) |  |
| Histological grade |  |  | 0.674 |  |  | 0.526 |
| I | 2(14.3%) | 8(21.6%) |  | 1(6.7%) | 1(16.7%) |  |
| II | 7(50.0%) | 20(54.1%) |  | 8(53.3%) | 4(66.7%) |  |
| III | 5(35.7%) | 9(24.3%) |  | 6(40.0%) | 1(16.7%) |  |
| Stage |  |  | 0.346 |  |  | 0.072 |
| I-II | 11(78.6%) | 24(64.8%) |  | 6(40.0%) | 5(83.3%) |  |
| III-IV | 3(21.4%) | 13(35.2%) |  | 9(60.0%) | 1(16.7%) |  |
| ER |  |  | 0.002* |  |  | 0.856 |
| Positive | 4(28.5%) | 28(75.7%) |  | 4(26.7%) | 1(16.7%) |  |
| Negative | 10(71.4%) | 9(24.3%) |  | 11(73.3%) | 5(83.3%) |  |
| PR |  |  | 0.003* |  |  | 0.686 |
| Positive | 3(21.4%) | 25(67.6%) |  | 5(33.3%) | 2(33.3%) |  |
| Negative | 11(78.6%) | 12(32.4%) |  | 10(66.7%) | 4(66.7%) |  |
| HER-2 |  |  | 0.912 |  |  | 0.746 |
| Positive | 4(28.5%) | 10(27.0%) |  | 9(60.0%) | 4(66.7%) |  |
| Negative | 10(71.4%) | 27(73.0%) |  | 6(40.0%) | 2 (33.3%) |  |
| Ki67 status |  |  | 0.375 |  |  | 0.315 |
| <14% | 6(42.9%) | 21(56.8%) |  | 11(73.3%) | 3(50.0%) |  |
| ≥14% | 8(57.1%) | 16(43.2%) |  | 4(26.7%) | 3(50.0%) |  |
| Breast cancer subtypes |  |  | 0.040* |  |  | 0.744 |
| Luminal A | 3(21.4%) | 21(56.8%) |  | 6(40.0%) | 4 (66.7%) |  |
| Luminal B/HER-2 Neg | 0(0.0%) | 3(8.1%) |  | 2 (13.3%) | 1(16.7%) |  |
| Luminal B/HER-2 Pos | 1(7.1%) | 4(10.8%) |  | 3(20.0%) | 0(0.0%) |  |
| HER-2 | 3(21.4%) | 3(8.1%) |  | 2(13.3%) | 1(16.7%) |  |
| TNBC | 7(50.0%) | 6(16.2%) |  | 2(13.3%) | 0(0.0%) |  |

*P value < 0.05 was considered statistically significant

Abbreviation: LN (Lymph node), ER (Estrogen receptor), PR (Progesterone receptor), HER-2 (Human epidermal growth factor receptor-2)

**Table S4: Correlation of Notch3&IL6 and GATA-3&IL6** Expression in Patients with Breast Cancer

|  | Notch3low  (n=7) | Notch3high  (n=13) | P | r | GATA-3low  (n=5) | GATA-3high  (n=15) | P | r |
| --- | --- | --- | --- | --- | --- | --- | --- | --- |
| IL6 low | 3 | 12 | 0.015* | -0.545 | 2 | 12 | 0.037* | -0.467 |
| IL6 high | 4 | 1 |  |  | 3 | 3 |  |  |

*P value < 0.05 was considered statistically significant


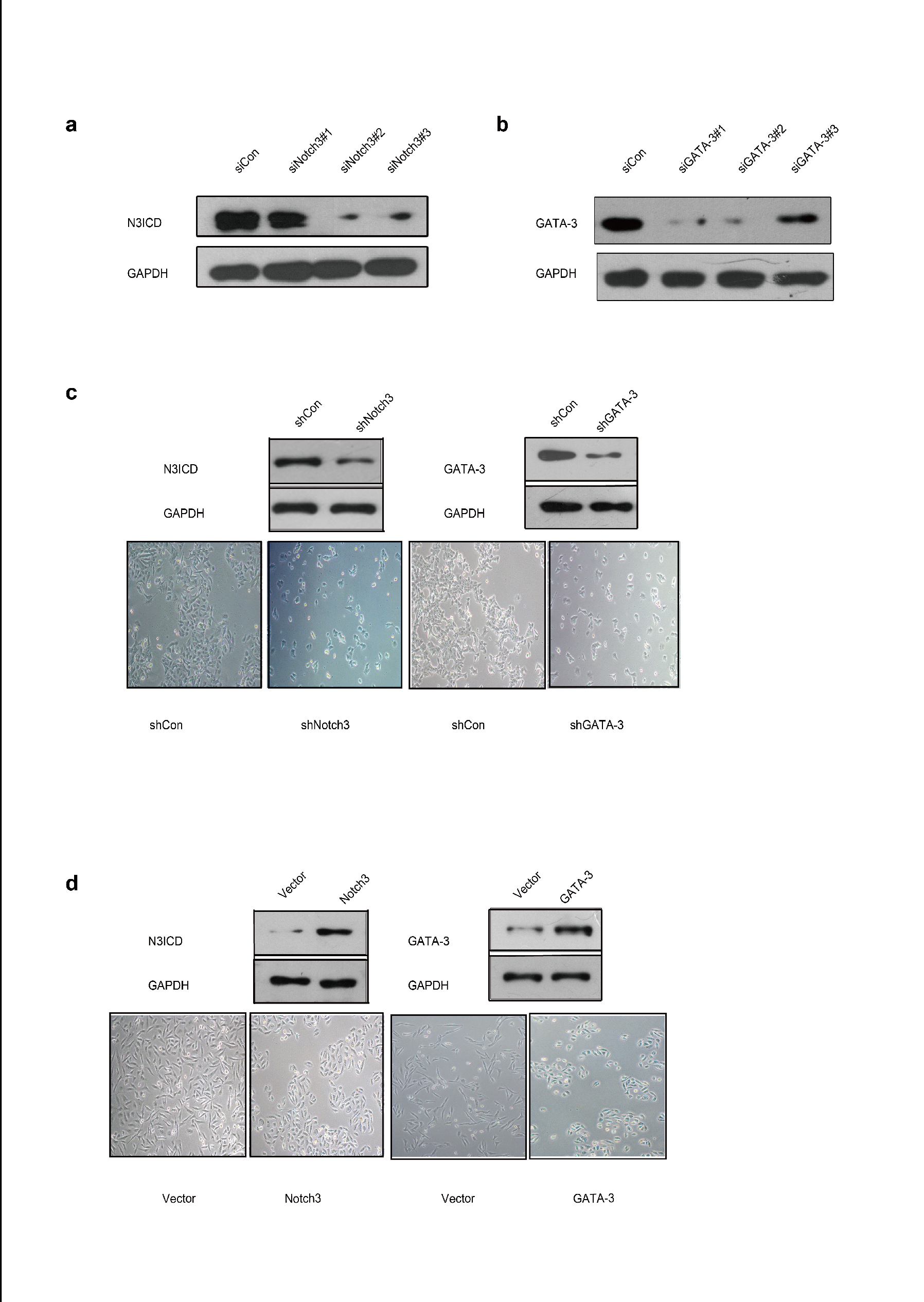


**Figure S1. Stably silencing Notch3 or GATA-3 changed Morphology of MCF-7 cells. (a)** Expression of Notch3 in MCF-7 cells analyzed by western blot when silencing by different sequence of siRNA. **(b)** Expression of GATA-3 in MCF-7 cells analyzed by western blot when silencing by different sequence of siRNA **(c)** Morphology of cells and expression of Notch3 or GATA-3 in MCF-7 analyzed by western blot when stably silencing Notch3 or GATA-3 by shRNA **(d)** Morphology of cells and expression of Notch3 or GATA-3 in MDA-MB-231 cells analyzed by western blot when overexpressing Notch3-ICD or GATA-3.


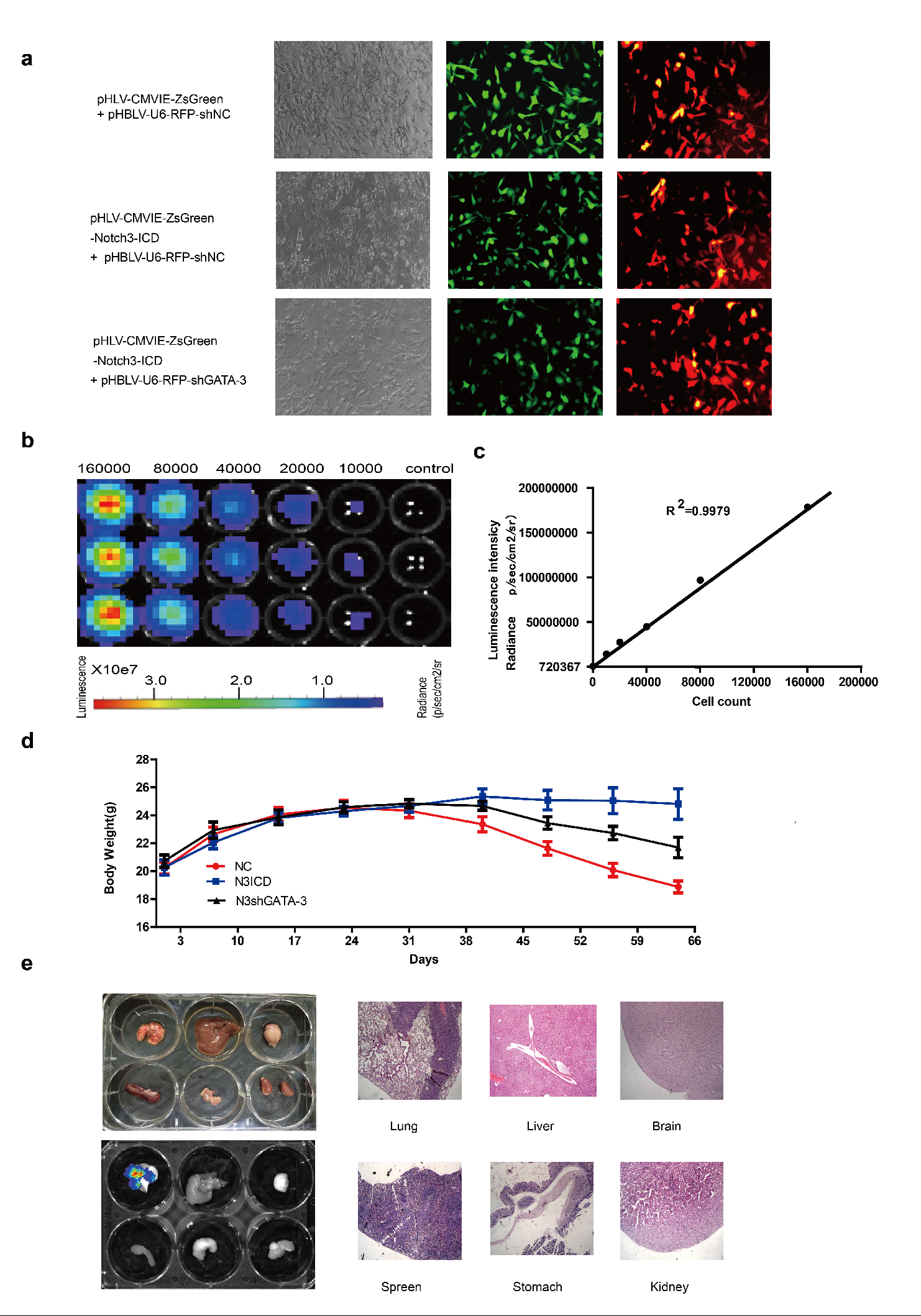


**Figure S2. Notch3 inhibits distant metastasis via regulation of GATA-3 in a Humanized Mouse Model**

Confocal fluorescent microscopy of MDA-MB-231 when stably overexpressing Notch3-ICD with or without knockdown of GATA-3 by lentivirus infection with different fluorescence. **(b-c)** The photon intensities of different cell numbers in the 3 groups are indicated (mean ±SEM). **(d)** A time course of mice body weight growth. Error bars show ±SEM. **(e)** Bioluminescence imaging of mice’s lung, liver, brain, stomach, spleen, kidney which were taken out within 5 mins after sacrifice (left). Representative hematoxylin and eosin staining for such organ in the 3 groups. (Amplification factor: 40X).


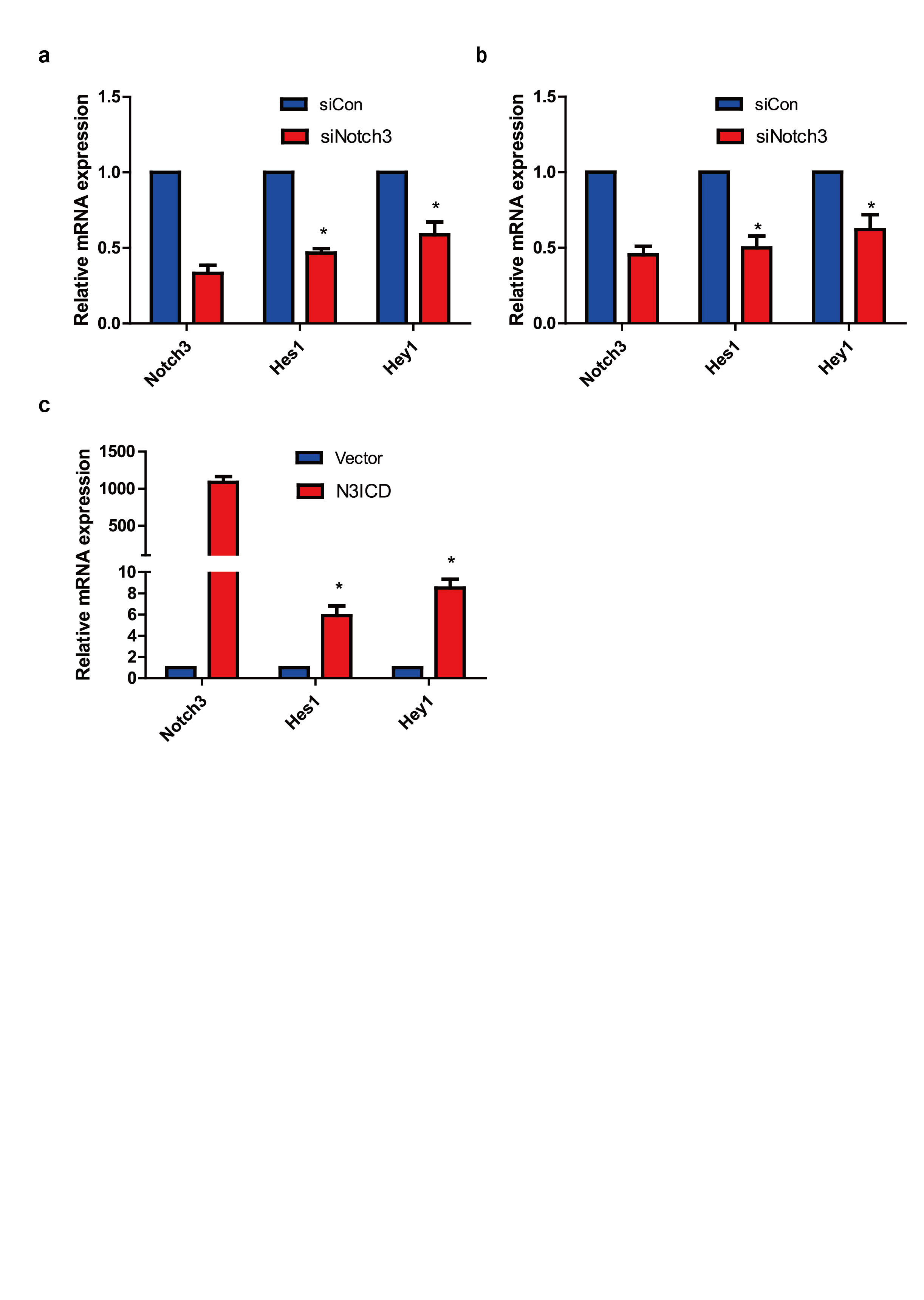


**Figure S3. Notch3 knockdown or N3ICD overexpression in breast cancer cells influenced downstream target Hes1 and Hey1**

**(a)** Expression of Notch3, Hes1 and Hey1 in MCF-7 cells analyzed by RT–PCR when silencing Notch3 by siRNA. **(b)** Expression of Notch3, Hes1 and Hey1 in T47D cells analyzed by RT–PCR when silencing Notch3 by siRNA. **(c)** Expression of Notch3, Hes1 and Hey1 in MDA-MB-231 cells analyzed by RT–PCR when overexpressing N3ICD.


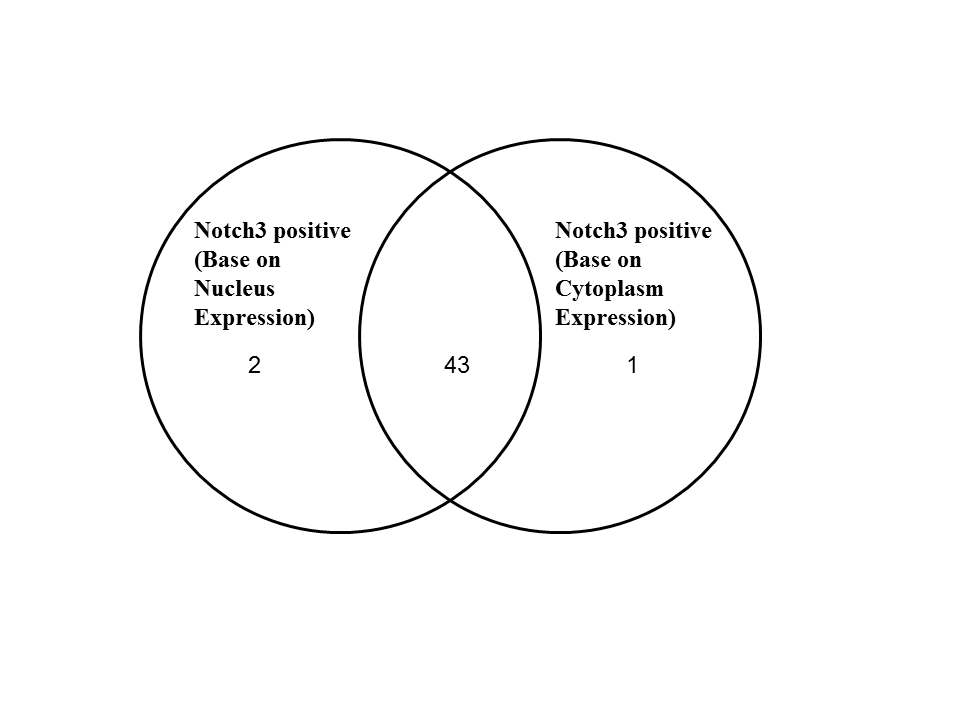


**Figure S4.** **Expression pattern of Notch3**

**
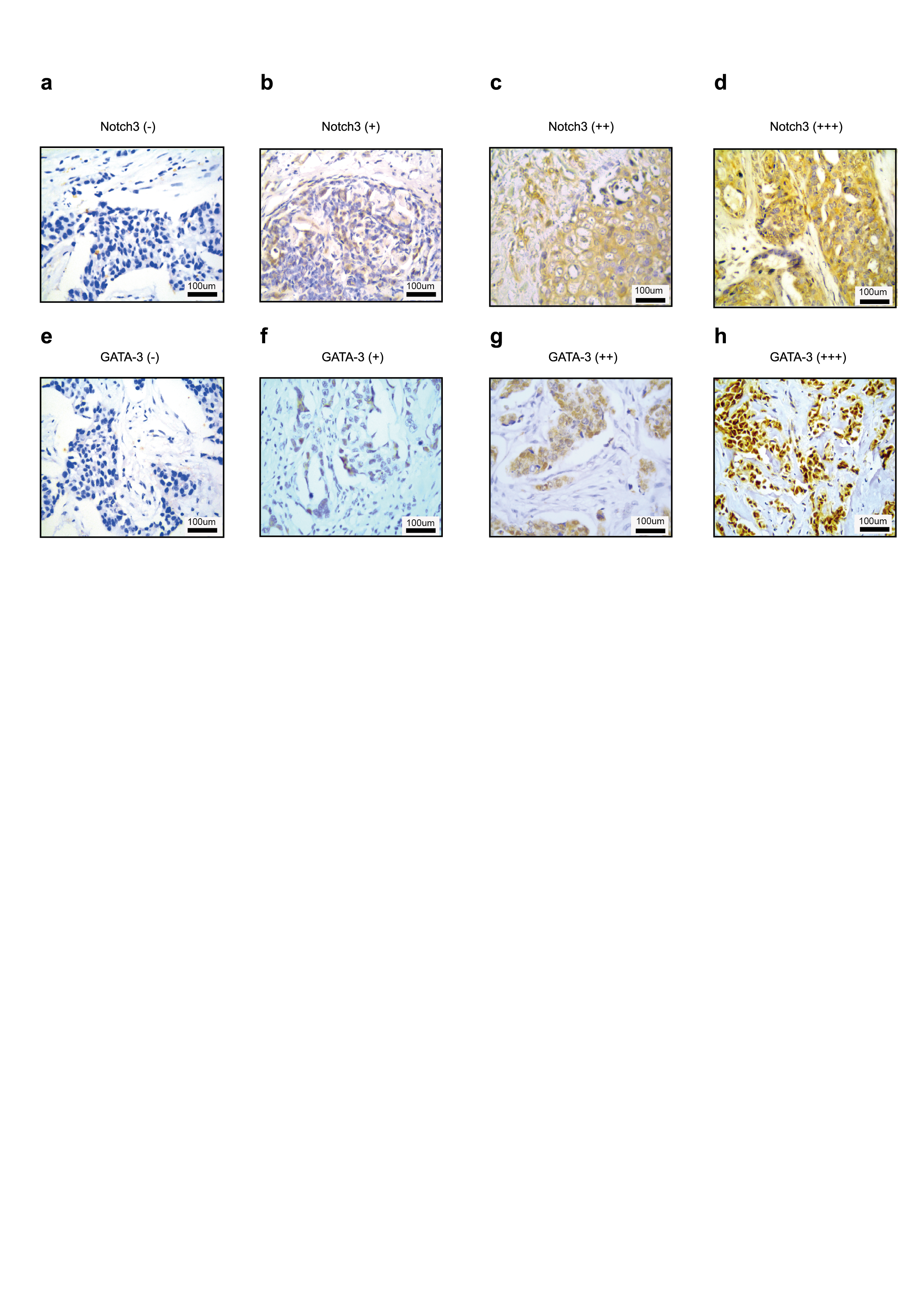
**

**Figure S5. Notch3 expression is associated with GATA-3 in breast cancer clinical cases.**

**(a–d)** Representative pictures of Notch3 with different expression levels, **(e–h)** Representative pictures of GATA-3 with different expression levels. Samples were stained by immunohistochemistry.
